# Trends in the incidence of hospital-treated suicide attempts during the COVID-19 pandemic in Oviedo, Spain

**DOI:** 10.1192/j.eurpsy.2023.6

**Published:** 2023-02-03

**Authors:** J. Fernandez-Fernandez, L. Jiménez-Treviño, E. Seijo-Zazo, F. Sánchez Lasheras, M. P. García-Portilla, P. A. Sáiz, J. Bobes

**Affiliations:** Faculty of Medicine and Health Sciences, University of Oviedo, Oviedo, Spain

**Keywords:** adolescent, COVID-19, lockdown, suicide attempt

## Abstract

**Background:**

The potential impact of the COVID-19 pandemic on suicidal behavior has generated predictions anticipating an increase in suicidal tendencies. The aim of this research is to study its influence on the incidence of hospital-treated suicide attempts throughout the year 2020 in Oviedo, Spain.

**Methods:**

Data were collected on all patients admitted to the emergency department of Central University Hospital of Asturias in Oviedo for attempted suicide during 2020. Incidence rates were calculated for three lockdown periods. Suicide attempt trends in 2020 were compared with a non-COVID-19 year (2009) to avoid seasonal variations bias. Chi-square and Fisher’s exact tests were performed. The influence of COVID-19 incidence in Oviedo was analyzed using Spearman’s correlation coefficient.

**Results:**

The cumulative incidence rate of attempted suicide per 100,000 person-years was 136.33 (pre-lockdown), 115.15 (lockdown), and 90.25 (post-lockdown) in adults (over 19 years old), and 43.63 (pre-lockdown), 32.72 (lockdown), and 72.72 (post-lockdown) in adolescents (10–19 years old). No association was found with COVID-19 incidence rates (Spearman’s rho −0.222; *p* = 0.113). Comparing the years 2020 and 2009, statistically significant differences were observed in adolescents (Fisher’s exact test; *p* = 0.024), but no differences were observed in adults (chi-square test = 3.0401; *p* = 0.218).

**Conclusions:**

Hospital-treated suicide rates attempted during the COVID-19 outbreak in Oviedo, Spain showed a similar trend compared with a non-COVID-19 year. In contrast, the number of adolescents hospital-treated for attempted suicide increased during lockdown, suggesting more vulnerability to COVID-19 restrictions after the initial lockdown period in this age group.

## Introduction

On March 11, 2020, the World Health Organization declared COVID-19 a global pandemic [1]. In the wake of that declaration, on March 15, a state of emergency was decreed in Spain, establishing measures including a declaration of “lockdown” for the entire population. The impact of this pandemic on general health and more specifically on mental health over that year is one of the main public health challenges we have faced [2]. Both the direct impact of the disease itself and the consequences of the implemented measures have resulted in an increase in problems such as social isolation, economic crisis, unemployment, fear of contagion, and loss of loved ones [3, 4]. All these factors may have a negative influence on mental health and are among the primary risk factors for conditions involving anxiety, suicidal behavior, and substance abuse [5–7]. All of this has led to an increase in pandemic-related psychological discomfort, with increases in depressive symptoms, insomnia, alcohol consumption, and anxiety problems [8–10]. This impact can be observed both in the general population and in patients who were already suffering from mental illnesses, the latter being more vulnerable [11]. Despite all this, it is interesting to point out that, in certain people, the effect of pandemic on mental health has not been negative or has even been positive. This was observed in a Danish trial with previous depression [12] as well as in a cohort of young adults (22 years old) in Quebec, in which no change in depression and anxiety levels was reported [13].

As a result of the foregoing, several authors expected an increase in suicidal behaviors in 2020 [14]. Although there is growing evidence on the subject, the quantity and quality of studies on pandemic-related suicidal behavior are still limited, in particular with regard to the Spanish population. Furthermore, the results of existing studies are disparate and contradictory. With respect to suicide rates, large population studies focusing on high-income countries over the first 6 months of the pandemic show that, in most cases, not only has there been no increase in suicide rates, but that there has actually been a decrease in most countries [15]. With respect to the European population, in central areas such as Austria, in the first 6 months of the pandemic, there was a decrease in suicide deaths [16], a trend also observed in Italy [17] and Portugal [18]. In other countries like Germany, the data were similar to previous years [19]. Although there are several publications at the European level, those present only data from the first few months of the pandemic. Data from the full year in a Japanese study show an increase in suicide rates in the second half of the year [20].

Regarding suicide attempts, there are several studies that analyze the trend throughout the year. In the meta-analysis by Dubé published in 2021, which includes data from 52 different countries, an increase was found in suicidal behavior, with an increase in suicidal ideation and suicide attempts, especially in the female population and in young males [21]. These data are consistent with a Spanish study (in adults over 18) with regard to suicidal ideation [22] but not suicide attempts [23]. Likewise, a study on psychiatric admissions to a Swiss emergency department did not show an increase in individuals admitted to the emergency service for attempted suicide [24]. However, a study on psychiatric inpatients in Italy reported an increase in suicide attempts in this population [25].

It should be noted that adolescents, as a particularly vulnerable group, have also been affected by the pandemic. Several articles show how the incidence of suicidal behaviors in adolescents (including suicide attempts) increased after lockdown, especially in the female population [26, 27].

The main objective of this study is to analyze trends in hospital-treated suicide attempts as they relate to COVID-19 and lockdown restrictions by collecting information on all people admitted to the emergency department of the Central University Hospital of Asturias (HUCA) for attempted suicide throughout the year 2020, exploring possible age and sex differences. These data will be compared with data prior to the pandemic in order to rule out seasonal influences.

## Methods

### Setting

Asturias is one of 17 autonomous communities in Spain. It is located on the north coast of Spain and has a population of just over 1 million. The study was conducted at HUCA, the university hospital located in Oviedo, the capital city of Asturias. HUCA is the referral hospital for Oviedo and its surrounding municipalities, which constitute Health Area IV of Asturias, and this was considered the study catchment area. According to the 2020 Asturian Health Portal population estimate, Health Area IV of Asturias had a population of 331,676.

### Case definition and ascertainment

Suicidal presentations were classified as suicide attempts using the definition devised by the working group of the former WHO/European Multicentre Study on Suicidal Behavior [28]: “An act with nonfatal outcome, in which an individual deliberately initiates a non-habitual behavior that, without intervention from others, will cause self-harm, or deliberately ingests a substance in excess of the prescribed or generally recognized therapeutic dosage, and which is aimed at realizing changes which the subject desired via the actual or expected physical consequences.”

The method of the suicide attempt was recorded using the International Statistical Classification of Diseases and Related Health Problems 10th Revision (ICD-10) codes. Alcohol was included as a suicide method if justified by the patient’s statement of the methods used in the attempt.

All patients admitted to the emergency department of HUCA in Oviedo for a suicide attempt during the 12-month period from January 1, 2020 to December 31, 2020 were examined. Those meeting the case-definition criteria were selected on admission to the emergency department by a psychiatrist trained to understand and apply the case definition. Sociodemographic and clinical information required for the follow-up form was collected after discharge through the electronic medical record.

### Age groups

The sample was split into two age groups (10–19 and over 19 years) to compare suicide attempt trends between adolescents and adults. We used the 19-year-old cut-off point to match the age groups used in the Spanish National Institute of Statistics (INE) [29].

### Pandemic and lockdown periods

Lockdown in Spain was imposed from March 14, 2020 to June 21, 2020, thus defining three different periods of the pandemic: pre-lockdown (January 1 to March 13), lockdown (March 14 to June 21), and post-lockdown (June 22 to December 31).

### COVID-19 incidence rates

In addition, weekly data on COVID-19 cases were retrieved in order to analyze a possible influence.

### Previous suicide attempt trends

The incidence of suicide attempts can experience significant seasonal variations over the course of a year. For this reason, it is interesting to compare 2020 trends with data from previous years. We did not collect suicide attempt data in 2019, but we had data for a non-COVID-19 year from a previously published study on 2009 trends [30], which we used for this purpose.

### Statistical analysis

Suicide attempt rates per 100,000 person-years in 2020 were calculated for the three study periods. Hospital-treated suicide attempts in each period of the year 2020 were escalated as if that period had been 1 year long for easier comparison with other studies.

Population estimates for our catchment area were obtained from the Spanish National Statistics Institute [31], disaggregated by sex and age groups.

In calculating rates, only the first presentation of each patient was considered. Patients residing outside the catchment area who presented to hospital following an attempted suicide were included in the rate calculations to offset, to some extent, catchment area residents who may have presented elsewhere. Assuming that the number of persons who presented following attempted suicide (*x*) follows a Poisson distribution, 95% confidence intervals for the rates were calculated using the normal approximation, that is, confidence interval = (*x* ± 2*√x)*100,000/population.

Suicide attempt incidence rates were calculated with a 95% confidence interval.

COVID-19 incidence data on the different pandemic periods were taken from the Government of the Principality of Asturias website [32].

Spearman’s rank correlation coefficient was used to compare COVID-19 incidence rates with suicide attempt rates over the study period.

Spearman’s rank correlation coefficient was also performed adjusting for sex and age to search for a relationship among these variables.

Data from 2020 and 2009 were compared using a chi-square test in the adult group. Fisher’s exact test was performed in the adolescent group, as one or more cell counts of less than 5 were expected in the cross-tabulation table.

## Results

A total of 337 suicide attempt presentations (103 males [30.6%] versus 234 females [69.4%]) were recorded during the study period at the emergency department of HUCA in Oviedo, yielding a cumulative incidence of 102.47 suicide presentations/100,000 person-years. For the adolescent group, a total of 41 suicide attempts were recorded (*n* = 12 pre-lockdown, *n* = 8 during lockdown, and *n* = 21 post-lockdown). In adults, the total was *n* = 296 (*n* = 80 pre-lockdown, *n* = 86 during lockdown, and *n* = 130 post-lockdown).

The mean age (SD) of suicide presentations was 45.51 (18.32) for males and 43.33 (18.44) for females. There was no statistically significant difference in mean age between males and females (*p* = 0.145).

### Hospital-treated suicide attempts versus lockdown periods

Table 1 shows the hospital-treated suicide attempt rates in 2020 per 100,000 person-years in Health Area IV of Asturias during the lockdown periods. In this table, information is presented by age groups (10–19 and over 19 years) and by sex.

**Table 1.** Incidence of hospital-treated suicide attempts in 2020 by age group, sex, and pandemic period (suicide attempts/100,000 people/year [95% CI]).

Lockdown period	Age	*N*	Total rate	Total 95% CI		*N*	Male rate	Male 95% CI		*N*	Female rate	Female 95% CI	
Pre-lockdown (weeks 1–11)	All ages	92	132.24	119.82	144.66	32	117.06	98.42	135.70	60	191.41	169.16	213.67
	10–19	12	43.63	18.95	68.32	0	0.00	0.00	0.00	12	89.47	38.85	140.10
	Over 19	80	136.33	122.60	150.06	2	117.06	98.41	135.70	48	153.13	133.22	173.04
Lockdown (weeks 12–25)	All ages	95	107.29	96.10	118.48	29	69.19	56.13	82.25	66	141.53	123.82	159.23
	10–19	9	32.72	11.35	54.10	1	10.50	0.00	21.01	8	59.65	18.33	100.97
	Over 19	86	115.15	102.53	127.77	28	80.48	65.02	95.94	56	145.38	125.98	164.78
Post-lockdown (weeks 26–52)	All ages	150	96.93	77.72	116.14	42	51.96	40.64	63.28	108	120.09	103.78	136.40
	10–19	20	72.72	40.86	104.58	3	22.69	0.00	45.38	17	126.75	66.54	186.97
	Over 19	130	90.25	79.08	101.43	39	58.12	44.98	71.27	91	118.28	100.77	135.78

According to the results shown in the table, taking all patients into account, there was a global decrease in suicide attempt rates during the lockdown and post-lockdown periods.

When sex is taken into consideration, incidence rates in males and females followed a parallel distribution, with a progressive decline throughout the year that continued beyond the lockdown.

When we split the sample by age (adults over 19 and adolescents from 10–19), the results change. In the adolescent population, suicide attempt rates decreased during the lockdown but increased post-lockdown. This change in trend is due to the rate observed in young girls, which decreased during the lockdown, but dramatically increased post-lockdown, a situation that was not observed in young males.

### Hospital-treated suicide attempts versus COVID-19 incidence

**Figure 1.**
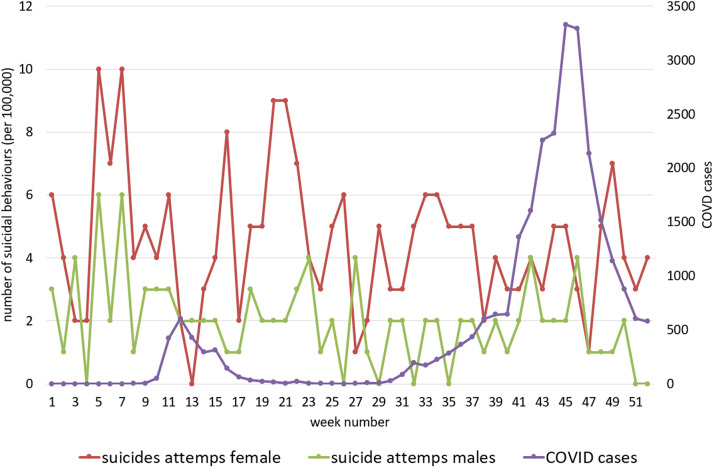
COVID-19 cases and hospital-attended suicide attempts at all ages by sex in 2020.

**Figure 2.**
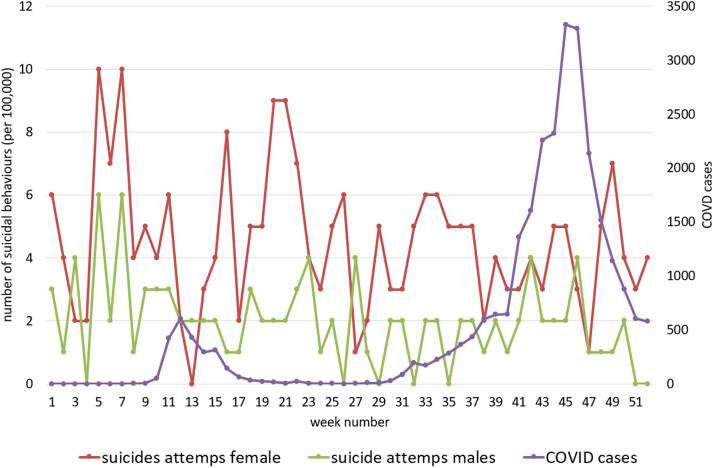
COVID-19 cases and hospital-attended suicide attempts in the over 19-year-old group by sex in 2020.

**Figure 3.**
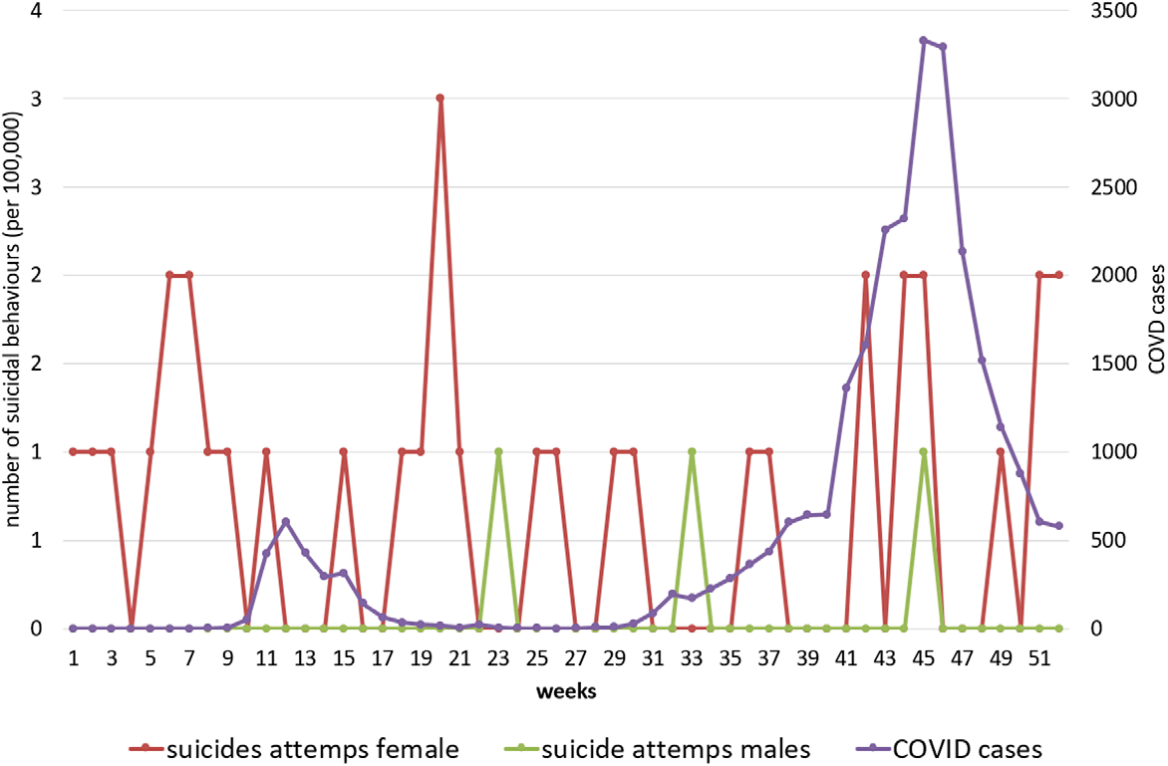
COVID-19 cases and hospital-attended suicide attempts in the 10–19-year-old group by sex in 2020.

Figures 1– 3 show COVID-19 and suicide attempt incidence curves in all ages and by age groups (adolescents and adults) by sex during 2020.

Spearman’s rank correlation coefficient did not show any statistical relationship between hospital-treated suicide attempts when compared with COVID-19 incidence in cases per week of individuals of all ages (Spearman’s rho −0.222; *p* = 0.113) or by sex: females (Spearman’s rho −0.240; *p* = 0.086) and males (Spearman’s rho −0.117; *p* = 0.407) (Figure 1).

Furthermore, there were no statistical relationships after splitting the sample by age groups and sex: all adults (Spearman’s test rho −0.215; *p* = 0.126); female adults (Spearman’s rho −0.240; *p* = 0.087); male adults (Spearman’s rho −0.157; *p* = 0.265) (Figure 2); all adolescents (Spearman’s rho 0.098; *p* = 0.490); female adolescents (Spearman’s rho −0.202; *p* = 0.150); and male adolescents (Spearman’s rho 0.145; *p* = 0.307) (Figure 3).

### 2020 versus 2009 hospital-treated suicide attempt trends by age group

**Figure 4.**
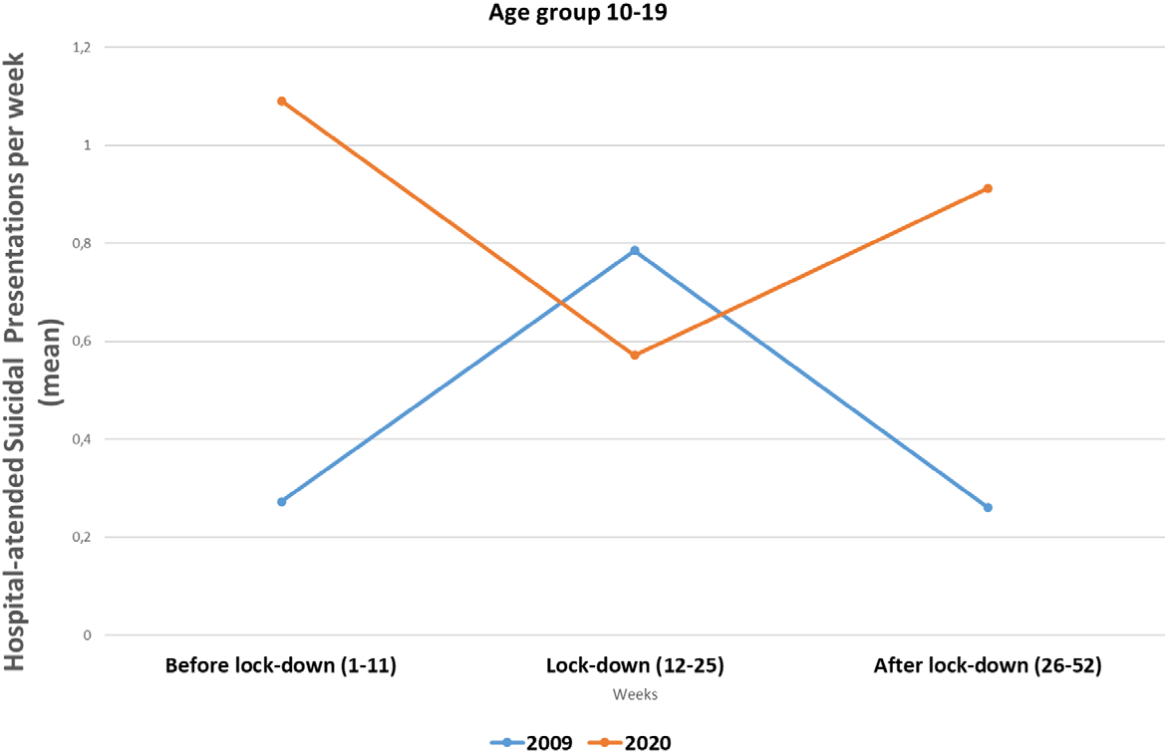
Mean of hospital-attended suicidal presentations per week comparing 2009 with 2020 in three periods of the year in 10–19 age group.

**Figure 5.**
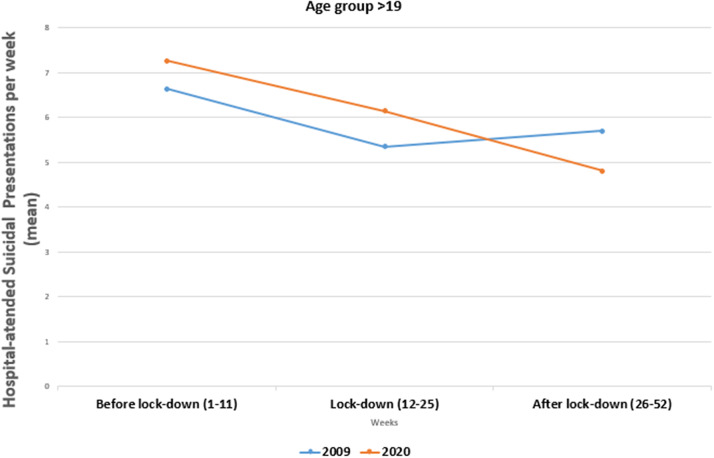
Mean of hospital-attended suicidal presentations per week comparing 2009 with 2020 in three periods of the year in >19 age group.

Statistically significant differences were observed in the 0–19-year age group. Fisher’s exact test was performed in the 10–19-year group because one of the cells had a value = 3 with *p* = 0.024. On the contrary, no changes were observed in the over 19-year age group. In this case, a chi-square test was used: *X* ^2^ = 3.040 and *p* = 0.218 (see Table 2 and Figures 4 and 5).

**Table 2.** Hospital-attended suicide attempts by age group comparing 2020 with 2009 during lockdown periods (in weeks).

	2009		2020	
	Age group 10–19 (*n*)	Age group > 19 (*n*)	Age group 10–19 (*n*)	Age group > 19 (*n*)
Before lockdown (1–11)	3	73	12	80
Lockdown (12–25)	11	75	8	86
After lockdown (26–52)	6	154	21	130

*Note:* 2020 versus 2019 (10–19 years) Fisher’s exact test: *p* = 0.024; 2020 versus 2019 (>19 years) chi-square = 3.040; *p* = 0.219.

Figure 5 shows opposite trends in adolescents hospital-treated for suicide attempts in 2020 compared with 2009. The summer period had the highest hospital-treated suicide attempt rates in 2009 but the lowest in 2020.

## Discussion

In summary, the results of the study reflect a declining incidence in suicide rates in both males and females in the adult population that may be explained by seasonal variations. The comparison between the annual trends in 2020 and 2009 (a non-COVID-19 year) showed no statistically significant effects; therefore, the variation cannot be related to the pandemic.

On the other hand, suicide attempts in young people showed a different trend when compared with adults and the non-COVID-19 year. After an initial decrease in suicide attempt rates during lockdown, a rebound phenomenon was observed post-lockdown, especially in young girls.

COVID-19 incidence rates (published by the authorities on a daily basis) did not seem to affect suicidal behavior, as in all groups no relationship between COVID-19 incidence and suicide rates was found throughout the year 2020.

### Overall trends in suicide attempt rates

Despite the fact that our results align with published studies focusing on the early phases of the pandemic and showed a downward trend in suicide attempts seen in the emergency department [23, 33, 34], our data comparing 2020 with a non-COVID-19 year cannot confirm this association.

On the other hand, the Dubé et al. meta-analysis associates the pandemic with an increase in suicide attempts, although these are moderated by a country’s “democratization index,” such that more democratized countries (e.g., those in Western Europe) have fewer suicide attempts [21]. This is consistent with other articles published in European populations, which found an increase in suicide attempts only in the case of Italy [33].

It is interesting to point out that the previously mentioned studies do not use a comparison with non-COVID-19 years. Changes observed during the course of the year tend to be attributed to COVID-19 incidence or other pandemic-related conditions such as movement restrictions or lack of access to emergency services.

### Sex and age differences

It has been remarked that there are sex and age differences in clinical profiles of suicidal behaviors that require a tailored approach to suicidal behavior research [35].

When considering sex differences in the adult sample, females had higher rates of hospital-treated suicide attempts than males, as seen in most studies [21], with the same downward trend for both sexes.

When the analysis is performed based on age, the data diverge from the general population. For adolescents under 19 years of age, the downward trend persisted only during lockdown. After June, a rebound above the initial data was observed in hospital-treated suicide attempts. This is consistent with specific studies in minors, which found that suicide attempts rose as of the start of the lockdown, especially in the months of June and September [26]. In addition, the rise was predominantly due to increased incidence in the female adolescent population [27].

In adolescents, the observed results suggest that lockdown was a protective factor, perhaps because young people were able to spend more time with family and strengthen the relationship with their parents (which may be a protective factor against suicide). A study published in 2020 conducted on a Chinese population evaluated symptoms of anxiety and depression in children and adolescents during lockdown [36]. Their results point to a worsening in the mental health of adolescents and children due to lockdown, although they do not compare the data with previous years. It is therefore difficult to establish a causal relationship with the lockdown. It is interesting to point out that, in this study, teens who were unsupervised on weekdays were more likely to be depressed and anxious. This would reinforce the idea of the family as a support for adolescents during this period.

Another possible explanation is the cancellation of in-person classes and the reduction in academic demands. As a result of the lockdown, in-person classes in Asturias were suspended and only online classes were taught. For teens, socializing can be challenging and a stress-generating factor. This may explain why in September, with the resumption of in-person classes and the progressive reopening of extracurricular activities, there was an increase in teens seen in the emergency department increased for attempted suicide.

In addition, preventive measures focused especially on the adult population and the elderly, without considering the vulnerability of adolescents. Another significant fact is that attempts increased in September. This may be related to the start of classes and the increase in stressors, academic load, and social demands. For minors with scholastic and adjustment problems, lockdown could have meant a decrease in perceived stress.

The results we have observed in the incidence of hospital-treated suicide may reflect not only trends in emergency department utilization, but the impact on overall suicidal behaviors. This is because we now have data from the Central University Hospital of Asturias in Oviedo showing a steep increase in suicidal-behavior-related admissions in the Adolescent Psychiatric Hospitalization Unit between 2020 and 2019. In the post-COVID-19 period of 2020, 63% of the admissions were related to suicidal behavior compared with 34% in the pre-pandemic year, and self-harm behaviors were present in 51.9% of the admissions post-COVID-19 compared with 7.4% in the previous year [37]. In contrast to adolescents [24], the increase in risk factors and psychopathology throughout 2020 did not translate into an increase in suicide attempts in the adult population. In a recent study published in Cataluña [38], the total number of patients admitted for suicidal behavior decreased relative to the previous year, a finding consistent with our sample.

Similar trends have been published in Spain with respect to the number of suicide deaths. The Spanish National Institute of Statistics reported a 100% increase in suicide deaths in adolescents in 2020, while overall figures in adults did not show significant variations [29].

### Limitations

The main limitations of this study include the small sample size in the adolescent group, which may result in a lack of statistical power. The collection of data at the emergency department of HUCA may have missed some suicide attempts. Although the suicide prevention protocol in the catchment area recommends psychiatric evaluation of all suicide attempts, less severe suicide attempts may have not been seen at the hospital. Moreover, our sample is a clinical emergency sample that may not be representative of the general population.

It is important to point out that it would have been desirable to compare our data with the years immediately prior to 2020. Although we compared our data with a non-COVID-19 year, the long gap in time between the two samples may have led us to erroneous conclusions. Trends in suicide attempts may have changed over 10 years.

## Conclusion

In conclusion, the present study reflects a decrease in hospital-treated suicide attempts in adult patients, with a constant downward trend throughout the year, but this trend cannot be attributed to the pandemic situation and may be explained by seasonal variations.

In young patients, a decrease in hospital-treated suicide attempts was observed during lockdown with a rebound in the post-lockdown period. In this age group, changes in trends may be attributed to the pandemic situation. With regard to sex, data also reflect greater vulnerability in young females. These results should be taken into account when developing future suicidal behavior prevention strategies aimed at the most vulnerable groups.

Trends in suicidal behavior should also be monitored for the year 2021, as suicidal behaviors may increase over time after natural disasters [39].
